# Supplementary material for: The role of the coherence in the cross-correlation analysis of diffraction patterns from two-dimensional dense mono-disperse systems
Source: Sci Rep. 2015 Nov 18;5:16573. doi: 10.1038/srep16573 (PMC4649615; doi:10.1038/srep16573)
Supplement: Supplementary Information [file srep16573-s1.doc]

**The role of the coherence in the cross-correlation analysis of diffraction patterns from two-dimensional dense mono-disperse systems**

T. Latychevskaia^1^, G. F. Mancini^2^ and F. Carbone^2^*

**Supplementary Note**

**Code1:**

Calculation of the cross-correlation function of the azimuthal intensity distribution

% This code finds the centre of a diffraction pattern, extracts the azimuthal

% intensity distribution (I(phi)) at a selected S0 vector value,

% and calculates its cross-correlation function by employing the formula

% CCF(D) = (<I(S0,phi)I(S0,phi + D)> - <I(S0,phi)>^2)/<I(S0,phi)>^2

% as defined by Wochner et al. in "X-ray cross correlation analysis

% uncovers hidden local symmetries in disordered matter", PNAS 106,

% 11511–11514 (2009).

% The code is written by Tatiana Latychevskaia, 2015.

% Citation for this algorithm:

% Tatiana Latychevskaia, Giulia F. Mancini and Fabrizio Carbone,

% "The role of the coherence in the cross-correlation analysis of

% diffraction patterns from two-dimensional dense mono-disperse systems"

% Scientific Reports 5, XXX (2015).

% Inputs

% dp diffraction pattern in TIFF format,

% S0 coordinate of S-vector value at which the intensity should

% be extracted, in pixels,

% Delta range (S0-Delta:S0+Delta) in which S-vector values are

% averaged, optimal value is 1-3 pixels,

% step step-size for extracting S-vector values in the range

% (S0-Delta:S0+Delta.)

% Outputs

% Iphi azimuthal intensity distribution at S0

% CCF1 cross-correlation function of azimuthal intensity at S0.

%%%%%%%%%%%%%%%%%%%%%%%%%%%%%%%%%%%%%%%%%%%%%%%%%%%%%%%%%%%%%

close all

clear all

%%%%%%%%%%%%%%%%%%%%%%%%%%%%%%%%%%%%%%%%%%%%%%%%%%%%%%%%%%%%%

% reading diffraction pattern from TIFF file

dp = im2double(imread('diffraction_pattern.tif'));

[N, N1] = size(dp);

%%%%%%%%%%%%%%%%%%%%%%%%%%%%%%%%%%%%%%%%%%%%%%%%%%%%%%%%%%%%%

% finding the centre of the diffraction pattern by finding the maximum

% of smoothed (low-pass filtered) diffraction pattern.

h = [1 1 1 1 1

1 1 1 1 1

1 1 4 1 1

1 1 1 1 1

1 1 1 1 1];

dp_sm = imfilter(dp,h);

centermax = max(max(dp_sm));

[x0, y0] = find(dp_sm == centermax);

%%%%%%%%%%%%%%%%%%%%%%%%%%%%%%%%%%%%%%%%%%%%%%%%%%%%%%%%%%%%%

% extracting the azimuthal distribution of intensity at S0 via

% transformation from Cartesian to polar coordinates is done

% by averaging the intensity in the range S0-Delta to S0+Delta,

% where Delta is 1-3 pixels. This allows compensating for intensity

% variation between neighbouring pixels due to experimental noise

% and obtaining a smooth intensity distribution.

Delta = 3; % range (S0-Delta:S0+Delta)

step = 0.2; % step size for calculating from (S0-Delta) to (S0+Delta)

Nphi = 360; % number of sampling points of the azimuthal distribution

S0 = 185; % selected S-coordinate

Iphi = zeros(1,Nphi);

for S = S0-Delta:step:S0+Delta

x = zeros(Nphi,1);

y = zeros(Nphi,1);

for ii = 1:Nphi

phi(ii) = ii-1;

x(ii) = round(x0 + S*cos(phi(ii)*pi/180));

y(ii) = round(y0 + S*sin(phi(ii)*pi/180));

IphiS(ii) = dp(x(ii),y(ii));

end

Iphi = IphiS + Iphi;

end

k = 2*Delta/step + 1;

Iphi = Iphi/k;

%%%%%%%%%%%%%%%%%%%%%%%%%%%%%%%%%%%%%%%%%%%%%%%%%%%%%%%%%%%%%

% calculating the cross-correlation function according to the formula

% CCF(D) = (<I(S0,phi)I(S0,phi + D)> - <I(S0,phi)>^2)/<I(S0,phi)>^2

B = (sum(Iphi(:))/Nphi)^2;

CCF0 = real(ifft((abs(fft(Iphi))).^2))/Nphi;

CCF1 = zeros(1,Nphi);

CCF1(:) = (CCF0(:) - B)/B;

%%%%%%%%%%%%%%%%%%%%%%%%%%%%%%%%%%%%%%%%%%%%%%%%%%%%%%%%%%%%%

%% plot of the intensity distribution.

figure('Position',[1000 100 800 400])

axes('FontSize',14)

plot(Iphi,'LineWidth',2);

grid on

xlabel('azimuthal angle \it{\phi} / degree','fontsize',18)

ylabel('Intensity / a.u.','fontsize',18)

xlim([0 360])

h = legend(strcat('Azimuthal intensity at S=', num2str(S0), ' pixels'), 2);

set(h,'Interpreter','none','fontsize',14)

%%%%%%%%%%%%%%%%%%%%%%%%%%%%%%%%%%%%%%%%%%%%%%%%%%%%%%%%%%%%%

% plot of the CCF distribution.

set(figure,'Position',[1000,600,800,400]);

axes('FontSize',16)

plot(CCF1,'LineWidth',2);

grid on

xlabel('\Delta / degree','fontsize',18)

ylabel('CCF / a.u.','fontsize',16)

xlim([0 360])

h = legend(strcat('CCF at S=', num2str(S0), ' pixels'), 2);

set(h,'Interpreter','none','fontsize',14)

%%%%%%%%%%%%%%%%%%%%%%%%%%%%%%%%%%%%%%%%%%%%%%%%%%%%%%%%%%%%%

%%%%%%%%%%%%%%%%%%%%%%%%%%%%%%%%%%%%%%%%%%%%%%%%%%%%%%%%%%%%%

%%%%%%%%%%%%%%%%%%%%%%%%%%%%%%%%%%%%%%%%%%%%%%%%%%%%%%%%%%%%%

Code2:

Calculation of diffraction pattern of spherical particles at partial coherence

% This code simulated diffraction pattern of a few spheres of 10 nm in

% diameter at partial coherence;

% The code is written by Tatiana Latychevskaia, 2015

% Citation for this algorithm:

% Tatiana Latychevskaia, Giulia F. Mancini and Fabrizio Carbone,

% "The role of the coherence in the cross-correlation analysis of

% diffraction patterns from two-dimensional dense mono-disperse systems"

% Scientific Reports 5, XXX (2015).

% Inputs

% N pixel number,

% S0 object area size in nm,

% D diameter of spherical nanoparticle in nm

% Lcoh coherence length in nm

% Outputs

% ff_s far-field distribution for single particle

% dp_s diffraction pattern of single particle

% dp diffraction pattern of the entire sample at partial coherence

%%%%%%%%%%%%%%%%%%%%%%%%%%%%%%%%%%%%%%%%%%%%%%%%%%%%%%%%%%%%%

close all

clear all

%%%%%%%%%%%%%%%%%%%%%%%%%%%%%%%%%%%%%%%%%%%%%%%%%%%%%%%%%%%%%

% parameters, all lengths are in nm

N = 500; % number of pixels

S0 = 500; % area size in object domain in nm

D = 5; % diameter of spherical nanoparticle in nm

r = D/2; % radius of spherical nanoparticle in m

Lcoh = 10; % coherence length in nm

%%%%%%%%%%%%%%%%%%%%%%%%%%%%%%%%%%%%%%%%%%%%%%%%%%%%%%%%%%%%%

deltas = 2*pi/S0; % pixels size in s-coordinates

delta0 = S0/N; % pixels size in object plane

%%%%%%%%%%%%%%%%%%%%%%%%%%%%%%%%%%%%%%%%%%%%%%%%%%%%%%%%%%%%%

% Diffraction pattern of a single sphere equivalent to diffraction on round

% aperture. The distribution of the wavefront in the far-field is given by:

% U(X,Y) = (-i/(lambda*z))*2d-integral exp(-2*pi*i/(lambda*z)(x*X+y*Y))dxdy

% This problem has analytical solution:

% U(s) = (-i*2*pi*r^2/(lambda*z))*J(r*s)/(r*s)

% where J is the Bessel function of 1st kind, r is the radius of the sphere,

% s is coordinate in the far-field, s=sqrt(sx^2+sy^2),

% sx=2*pi*X/(lambda*z), sy=2*pi*Y/(lambda*z).

% Diffraction pattern is found as square of far-field distribution U(s)

s = zeros(N,N); % distribution of s-vector value

ff_s = zeros(N,N); % far-field distribution for single particle

for ii=1:N

for jj=1:N

sx = (ii-N/2-1)*deltas;

sy = (jj-N/2-1)*deltas;

s(ii,jj) = sqrt(sx^2+sy^2);

if (s(ii,jj) > 0)

ff_s(ii,jj) = besselj(1,r*s(ii,jj))/(r*s(ii,jj));

end

if (s(ii,jj) == 0)

ff_s(ii,jj) = 0.5;

end

end

end

f = 2*pi*r^2/(delta0^2);

ff_s = f*ff_s;

dp_s = ff_s.^2; % diffraction pattern of single particle

%%%%%%%%%%%%%%%%%%%%%%%%%%%%%%%%%%%%%%%%%%%%%%%%%%%%%%%%%%%%%

% Here as example, 10 particles separated by 7 nm and organized into

% triangular lattice are considered. The coordinates of particles in nm:

x = [-7 0 7 -10.5 -3.5 3.5 10.5 -7 0 7];

y = [6.06 6.06 6.06 0 0 0 0 -6.06 -6.06 -6.06];

%%%%%%%%%%%%%%%%%%%%%%%%%%%%%%%%%%%%%%%%%%%%%%%%%%%%%%%%%%%%%

ff = zeros(N, N); % complex-valued distribution in far-field

[size_x1,size_x2] = size(x);

for p = 1:size_x2

[new_size_x1,new_size_x2] = size(x);

% calculating interference term only if there are more than two particles

% in array;

if (new_size_x2 > 1)

x1 = x(1); % reading out the coordinates of the first particle;

y1 = y(1);

x(1) = []; % removing the coordinates of the first particle from array;

y(1) = []; % of the particle coordinates;

for ii = 1:new_size_x2-1

a = sqrt((x1 - x(ii))^2+(y1 - y(ii))^2);

% s (in nm) is the distance between the first particle and the current

% particle;

if (a < 4*Lcoh)

mu = exp(-a^2/(2*Lcoh^2));

x1p = N/2 + x1/delta0; % coordinate in pixels

y1p = N/2 + y1/delta0; % coordinate in pixels

xp = N/2 + x(ii)/delta0; % coordinate in pixels

yp = N/2 + y(ii)/delta0; % coordinate in pixels

for jj=1:N

for kk=1:N

ff(jj,kk) = ff(jj,kk) + mu*2*cos(2*pi*((x1p - xp)*(jj - N/2) + (y1p - yp)*(kk - N/2))/N);

end % end of kk

end % end of jj

end % end of if

end % end of ii

end % end of if

end % end of p

% adding the self-interference terms, which is 1 for every particle and the

% total sum is the number of particles:

ff = ff + size_x2;

dp = ff.*dp_s;

imshow(rot90(dp), []);

%%%%%%%%%%%%%%%%%%%%%%%%%%%%%%%%%%%%%%%%%%%%%%%%%%%%%%%%%%%%%

%%%%%%%%%%%%%%%%%%%%%%%%%%%%%%%%%%%%%%%%%%%%%%%%%%%%%%%%%%%%%

%%%%%%%%%%%%%%%%%%%%%%%%%%%%%%%%%%%%%%%%%%%%%%%%%%%%%%%%%%%%%
